# Mitogenome of *Coprophanaeus ensifer* and phylogenetic analysis of the Scarabaeidae family (Coleoptera)

**DOI:** 10.1590/1678-4685-GMB-2020-0417

**Published:** 2021-08-09

**Authors:** Catarine Aragone de Albuquerque Mello, Igor Costa Amorim, Alexandre Freitas da Silva, Giuliene Rocha de Medeiros, Gabriel Luz Wallau, Rita de Cássia de Moura

**Affiliations:** 1 Universidade de Pernambuco, Instituto de Ciências Biológicas, Laboratório de Biodiversidade e Genética de Insetos, Recife, PE, Brazil.; 2 Departamento de Entomologia, Instituto Aggeu Magalhães - FIOCRUZ, Recife, Pernambuco, Brazil.

**Keywords:** Dung beetle, mitochondrial genome, phylogenomics

## Abstract

Several studies about the phylogenetic relationships of the Scarabaeinae subfamily (Coleoptera: Scarabaeidae) have been performed, but some phylogenetic uncertainties persist including the relationship and monophyly of different tribes and some genera. The aim of this study was to characterize the mitogenome of *Coprophanaeus ensifer* in order to establish its position within the Scarabaeidae family and to contribute to the resolution of some phylogenetic uncertainties. The mitogenome was sequenced on the Illumina HiSeq 4000, assembled using the Mitobim software and annotated in MITOS WebServer. The phylogenetic trees were reconstructed by Bayesian inference. The *C. ensifer* mitogenome is a molecule of 14,964 bp that contains the number and organization of the genes similar to those of most Coleoptera species. Phylogenetic reconstruction suggests monophyly of the tribe Phanaeini and supports the hypothesis that Coprini is a sister group of Phanaeini. The results also revealed the position of the tribe Oniticellini which is grouped with Onthophagini and Onitini. The geographic distribution of these species that form the most ancestral clade suggests with Scarabaeinae originated in Africa.

## Introduction

In the order Coleoptera, the family Scarabaeidae is particularly interesting due to its large adaptive radiation, as well as being formed by lineages with different feeding habits, including phytophagous, saprophagous and copro-necrophagous species (Gunter et al., 2016). The species of this family were grouped into 19 subfamilies (Smith, 2009); among these, Scarabaeinae are important because these beetles provide ecosystem services such as recycling of organic matter, biological control of agricultural pests, and secondary seed dispersal (Nichols et al., 2008). This subfamily is characterized by high species diversity, with about 6,200 species taxonomically grouped into 267 genera and 12 tribes (Tarasov and Génier, 2015). 

Most phylogenetic studies indicate that eight of the 12 Scarabaeinae tribes are monophyletic (Eucraniini, Eurysternini, Gymnopleurini, Oniticellini, Onitini, Phanaeini, Scarabaeini, and Sisyphini) and one is polyphyletic (Deltochilini) (Tarasov and Génier, 2015). Contradictory results have been described for the remaining tribes (Ateuchini, Coprini, and Onthophagini), with morphological and/or molecular analyses suggesting monophyly of these tribes, although they are frequently reported as being paraphyletic or polyphyletic (Villalba et al., 2002; Bai et al., 2011; Tarasov and Génier, 2015; Tarasov and Dimitrov, 2016 and references therein). Differences in the number of lineages have also been reported for Coprini, with descriptions of two or three lineages (Philips et al., 2004; Monaghan et al., 2007). 

The phylogenetic relationships of some Scarabaeinae tribes are also contradictory; for example, the relationships among the Deltochilini, Eurysternini, Onthophagini, Oniticellini, Onitini and Sisyphini tribes are frequently altered (Tarasov and Génier, 2015). The position of the tribe Phanaeini is also uncertain since it formed a sister group with the tribe Eucraniini in molecular studies (Ocampo and Hawks, 2006; Monaghan et al., 2007) and with the tribe Coprini in morphological studies (Philips et al., 2004). Furthermore, based on morphological analyses, Vaz-de-Mello (2007) showed that Phanaeini also formed a cluster with Eucraniini, Onitini, and Onthophagini.

In the tribe Phanaeini, phylogenetic uncertainties have also been described within the genus *Coprophanaeus* d’Olsoufieff, 1924 (Maldaner et al., 2018). Phylogenetic analyses based on molecular markers (mitochondrial COI, COII and 16S genes) indicated *Coprophanaeus* to be paraphyletic, since the species of this genus form a clade with *Diabroctis mirabilis* and *Sulcophanaeus faunus* (Maldaner *et al*., 2018). Within the genus *Coprophanaeus*, taxonomic uncertainty also exists regarding *C.* (*Megaphanaeus*) *ensifer* (Maldaner *et al*., 2018). Individuals of this species exhibit a large variation in size, color and geographic distribution, suggesting the presence of cryptic species (Maldaner *et al*., 2019).

Considering the phylogenetic and taxonomic uncertainties, more robust analyses (using larger datasets) of Scarabaeinae tribes, genera and species are necessary (Tarasov and Dimitrov, 2016). In view of the absence of recombination and exclusively maternal inheritance in animals, mitochondrial DNA is used for phylogeographic inferences (Boore and Brown, 1998), resolution of taxonomic uncertainties, and phylogenetic reconstructions (Vilstrup et al., 2011). Specific mitochondrial genes (Baca et al., 2017; Nolasco-Soto et al., 2017; Villastrigo et al., 2019), or the complete mitogenome can be used in such studies, conferring greater robustness to the analysis by providing a larger set of molecular data (Yuan et al., 2016; Nie et al., 2018). Based on the mitogenome, phylogenetic uncertainties were resolved in different taxonomic groups, including the beetle family Chrysomelidae (Nie *et al*., 2018).

The present study aimed to characterize the mitochondrial DNA of *C.* (*M.*) *ensifer* and to investigate the phylogenetic relationships within the family Scarabaeidae. In addition, this study provides molecular markers that can be used to test specific taxonomic hypothesis such as assessing the presence of cryptic species in *C.* (*M.*) *ensifer*.

## Material and Methods

### Collection of the biological sample

*Coprophanaeus* (*M.*) *ensifer* (Germar, 1821) specimens were collected in Aldeia, Camaragibe (8º1’18” S; 34º58’52” W), Pernambuco, Brazil, with pitfall traps baited with rotten meat. This collection was authorized by IBAMA/SISBIO through the permanent license of zoological material of the class Insecta (16278 - 1). The specimens were identified at the Laboratory of Biodiversity and Insect Genetics (LGBI), University of Pernambuco (UPE), using the identification key of the genus *Coprophanaeus* (Edmonds and Zídek, 2010) and by comparison with other specimens of the Entomological Collection of the University of Pernambuco (CEUPE), Laboratory of Biodiversity and Genetics of Insects (LBGI), Institute of Biological Sciences, University of Pernambuco, Pernambuco, Brazil (curator: Rita de Cássia de Moura). In addition, the identification was confirmed by the taxonomist Fernando Silva (UFPA). The specimens were sacrificed and stored in 100% alcohol until the time of DNA extraction. Only one specimen was used for this study. 

**DNA extraction and sequencing**

DNA was extracted from pronotal tissue of one specimen using the phenol-chloroform protocol of Sambrook and Russel (2001). Genomic sequencing was performed by Macrogen Inc. on an Illumina HiSeq 4000 platform using the following parameters: preparation of the genomic library using the TruSeq DNA PCR-Free kit (350-bp insert size), an average read length of 150 bp, and a paired-end sequencing approach.

**Characterization of the mitogenome of *Coprophanaeus* (*M.*) *ensifer***

The sequences obtained were first treated with the Trimmomatic program (Bolger et al., 2014) to remove sequences with a quality less than Q20. Next, the mitochondrial genome was assembled using the Mitobim software (Hahn et al., 2013). The mitogenome of *Sarophorus sp*. (GenBank: JX412735.1) (Scarabaeinae) was used as reference to bait the reads in the first steps of Mitobim analysis. The consensus sequence was uploaded to the MITOS WebServer for gene annotation of the *C.* (*M.*) *ensifer* mitogenome using the genetic code 05 - invertebrate.

### Phylogenetic analysis

The phylogenetic trees were reconstructed using two datasets: 1) complete nucleotide sequence of the mitogenome, except for the control region; 2) concatenated amino acid sequence of protein-coding genes of the mitogenome. In addition to the mtDNA of *C.* (*M.*) *ensifer*, we used the Scarabaeidae mitogenomes available at NCBI until September, 2019. These mitogenomes included species of Scarabaeinae (32 spp.), Melolonthinae (6 spp.), Rutelinae (3 spp.), Cetoniinae (3 spp.), Aphodiinae (3 spp.), and Dinastinae (1 spp.). Additionally, three species of the family Lucanidae were included as outgroups (*Prosopocoilus gracilis*, *Odontolabis cuvera fallaciosa*, *Dynodorcus curvidens hopei*), since this family is considered sister group to Scarabaeidae, according to molecular and morphological evidence (Grebennikov and Scholtz, 2004; Timmermans et al., 2016; Yang et al., 2018). These sequences were retrieved from the National Center for Biotechnology Information database (NCBI). The accession numbers are listed in Table S1. The resulting sequences were aligned using MAFFT and then treated with Gblock 0.91b using default parameters (Castresana, 2000).

For phylogenetic analysis, the GTR G+I and MtREV G+I+F substitution models were used for nucleotide and protein sequences, respectively. These models were selected based on Akaike’s information criterion (AIC) using jModelTest v. 2.1.4 (Darriba et al., 2012) and ProtTest v. 3.4.2 (Abascal et al., 2005). The trees were reconstructed by Bayesian inference on the CIPRES Science Gateway webserver using the following MCMC parameters: Number of generations of 10 million, sampling every 1,000 generations, four Markov chains, and burn-in of 20%. The tree was visualized and edited using Figtree v. 1.4.3 (Rambaut, 2016).

## Results and Discussion

### Characterization of the mitogenome

**Figure 1.**
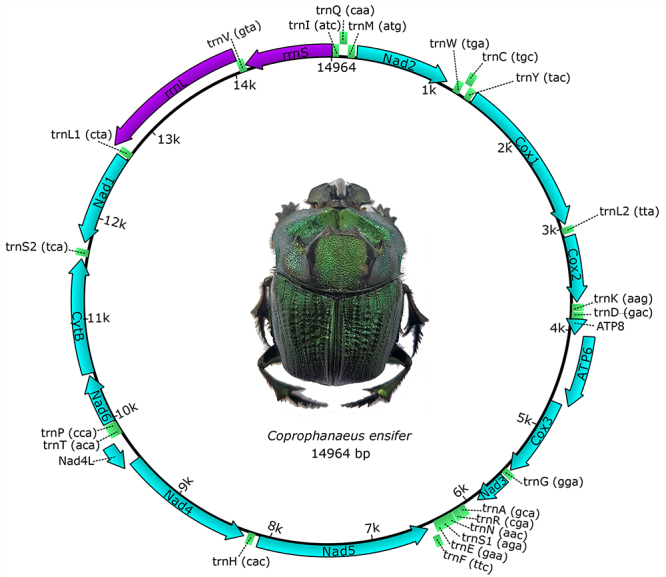
Organization genetic of *Coprophanaeus ensifer* mitogenome. Protein-coding genes, tRNA genes and rRNA genes are shown in blue, green and purple, respectively. The arrows indicate the direction of the genes.

The analysis of generated sequences allowed us to characterize the mitogenome of *Coprophanaeus* (*M.*) *ensifer*. A total of 1,190,241 reads were assembled in a mitogenome of 18,134 bp showing an average coverage depth of 9845x and average coverage breadth of 100%. Considering that the assembly and size of the molecule can be influenced by the repetitive nature of the control region (Gillett *et al*., 2014), the size of the molecule without the control region would be 14.964 bp (Figure 1) (accession number: MW122514 and Supplementary File S1). This is a common size when compared to the typical mitogenome of animals, with the complete mitochondrial genome of animals comprising 15 to 16 kb (Boore, 1999) and the control region in representatives of Coleoptera generally comprising 1 kb (Sheffield et al., 2008).

Mitogenome annotation of *C.* (*M.*) *ensifer* revealed two ribosomal RNAs (rRNA) and 22 transfer RNAs (tRNA), in addition to the following 13 protein-coding genes (CDS): ATP synthase membrane subunits 6 and 8 (ATP6 and 8), cytochrome b (CytB), cytochrome c oxidase 1 to 3 (COX 1-3), and NADH reductase subunits 1 to 6 (NAD1-6) and 4L (NAD4L) (Figure 1, Table 1). According to MITOS WebServer, 9 CDS and 14 tRNA are located on the positive strand (plus Strand), while 4 CDS, 2 rRNA and 8 tRNA are present on the negative strand (minus Strand) (Figure 1, Table 1). In addition, the positive strand has the lower content of guanine and thymine (G + T = 47%), indicating that this is the light strand (L), according to Lima and Prosdocimi (2017). The number, orientation and order of the genes are similar to those of most insect species (Cameron, 2014; Sheffield et al., 2009), as well as to those of the beetle species *Dichotomius schiffleri* (Amorim et al., 2017) and *Priasilpha obscura* (Sheffield *et al*., 2008). Within the family Scarabaeidae, they only differ from those observed in the genus *Cyphonistes* (Dynastinae), which possesses an inversion between the trnaA and trnaR genes, considered an apomorphy (Timmermans et al., 2016).

**Table 1.** Gene annotation in the mitogenome of *Coprophanaeus ensifer*.

Name	Start	Stop	Strand	Length
trnI (atc)	1	66	+	66
trnQ (caa)	64	132	-	69
trnM (atg)	151	219	+	69
Nad2	241	1149	+	909
trnW (tga)	1232	1299	+	68
trnC (tgc)	1292	1359	-	68
trnY (tac)	1361	1426	-	66
Cox1	1447	2961	+	1515
trnL2 (tta)	2986	3050	+	65
Cox2	3078	3716	+	639
trnK (aag)	3730	3801	+	72
trnD (gac)	3808	3874	+	67
ATP8	3875	4027	+	153
ATP6	4024	4689	+	666
Cox3	4698	5480	+	783
trnG (gga)	5491	5555	+	65
Nad3	5574	5903	+	330
trnA (gca)	6038	6105	+	68
trnR (cga)	6108	6174	+	67
trnN (aac)	6180	6247	+	68
trnS1 (aga)	6248	6316	+	69
trnE (gaa)	6317	6383	+	67
trnF (ttc)	6382	6448	-	67
Nad5	6456	8111	-	1656
trnH (cac)	8160	8226	-	67
Nad4	8257	9534	-	1278
Nad4L	9534	9788	-	255
trnT (aca)	9827	9892	+	66
trnP (cca)	9893	9959	-	67
Nad6	9971	10453	+	483
CytB	10478	11581	+	1104
trnS2 (tca)	11610	11677	+	68
Nad1	11739	12632	-	894
trnL1 (cta)	12651	12719	-	69
rrnL	12683	14053	-	1371
trnV (gta)	14051	14121	-	71
rrnS	14122	14964	-	843

Twenty-four intergenic spacers were identified in the mitogenome of *C.* (*M.*) *ensifer* (Table 1). Among these, the spacer between NAD1 and RNAtS2 is a common feature of the superfamily Scarabaeoidea (Jeong et al., 2020). However, this spacer is larger in *C.* (*M.*) *ensifer* (83 bp) (Table 1) compared to other Scarabaeoidea species which harbor a maximum spacer of 31 bp (e.g., *Eurysternus inflexus*) (Jeong *et al*., 2020). In the mitogenome assembled, this region had coverage depth around 7800x which is similar to the average whole mitogenome coverage depth (9845x), strongly supporting the presence of this region. 

The mtDNA of *C.* (*M.*) *ensifer* also contains four gene overlaps, totaling 46 bp (Table 1). Such overlaps have been reported in different Coleoptera species, including *Cheirotonus mansoni* (Shao et al., 2014) and *Amphimallon solstitiale* (Yang et al., 2019). This type of overlapping is related to the evolution of mtDNA, which tends to reduce its size over time due to selective pressure, causing a reduction in intergenic spacers that can accumulate in gene overlap (Schneider and Ebert, 2004; Sheffield et al., 2008, 2010). *C.* (*M.*) *ensifer*, selective pressure apparently did not cause major reductions in the mitogenome, since numerous and large intergenic spacers and only four gene overlaps were observed (Figure 1, Table 1). This number is smaller than that of other species of the genus (*Coprophanaeus* sp.) whose mitogenome possesses 12 gene overlaps.

The greatest gene overlap in *C.* (*M*) *ensifer* mtDNA is found between the tRNAL1 and rRNAL genes, corresponding to 37 bp of the 69 bp of this tRNA. Gene overlapping can lead to problems in the polycistronic transcription of mitogenomes (Sheffield et al., 2008, 2010), as it prevents the release of full-length RNAs of each overlapping gene from the same transcript (Boore, 1999). Considering this, each transcript of *C.* (*M*) *ensifer* mitogenome will produce only one functional tRNAL1 or rRNAL molecule. However, this effect may be minimized due to the presence of a second gene for leucine tRNA (tRNAL2), as proposed for the grasshopper *Rhammatocerus brasiliensis* (Amorim et al., 2020). In addition, an overlap between the atp6 and atp8 genes was observed, which is common in metazoans (Campbell and Barker, 1999) and is probably a plesiomorphic feature of this group.

### Phylogenetic analysis

**Figure 2.**
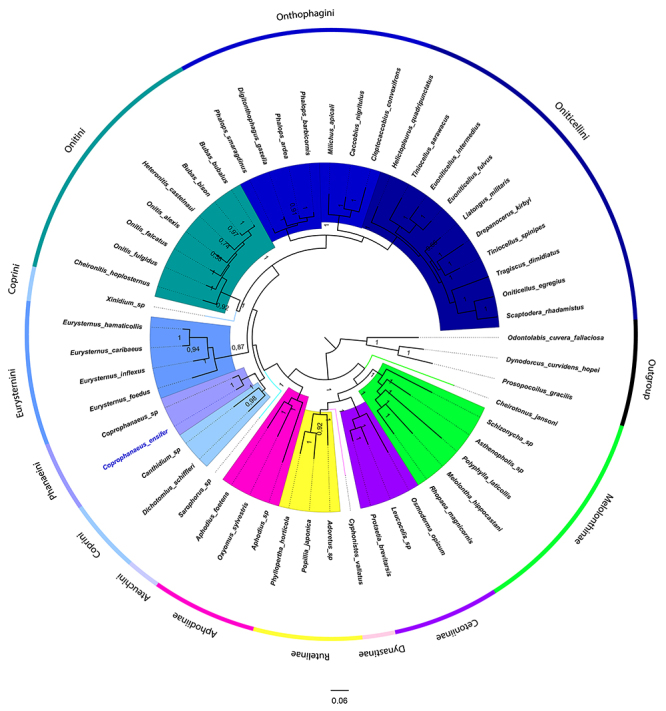
Phylogenetic reconstructions of the family Scarabaeidae based on mitochondrial protein sequences. These reconstructions were performed by Bayesian inference using the MtREV G+I+F substitution model. The colors of each clade distinguish the Scarabaeidae subfamilies as well as Scarabaeinae tribes. *Coprophanaeus ensifer* is highlighted in blue in the phylogeny.

Among the different subfamilies analyzed in the present study, only the subfamily Melolonthinae was found to be paraphyletic (Figure 2 and Figure S1). This result agrees with other phylogenies obtained based on the mitogenome (Song and Zhang, 2018). Specifically in the subfamily Dynastinae, only the mitogenome of *Ciphonistes vallatus* has been sequenced and it was therefore not possible to confirm whether this group is monophyletic, as suggested in molecular studies using mitochondrial (16S rRNA, 12S rRNA, and COI) and nuclear markers (28S, LSU, and rRNA) (Gunter et al., 2016). 

The analysis of protein and nucleotide sequences resulted in different phylogenetic relationships between the Scarabaeidae subfamilies (Figure 2, Figure S1 and S2). Such differences have been reported in several studies, including those on the family Scarabaeidae (Jeong et al., 2020). Nucleotide analysis revealed the topology (Dynastinae (Cetoniinae (Melolonthinae (Rutelinae (Aphodiinae + Scarabaeinae))))) (Figure 2). On the other hand, the results of protein analysis showed the topology (Melolonthinae (Cetoniinae (Rutelinae + Dynastinae))) (Scarabaeinae + Aphodiinae). The tree topology based on amino acid sequences was similar to that described in other studies (Song and Zhang, 2018). Considering this topology and the higher resolution at the nodes, the use of amino acid sequences is suggested for the phylogenetic placement of Scarabaeidae. This is contrary to the hypothesis of Cameron (2014) that states the loss of phylogenetic signal for amino acid sequences in taxonomic groups within the class Insecta.

Among the seven tribes analyzed of the subfamily Scarabaeinae, only Eurysternini, Onitini, Oniticellini and Phanaeini were monophyletic by the two approaches (Figure 2, Figure S1). The same result was reported for molecular analyses based on nuclear (28S) and mitochondrial genes (COI and rrnL) (Monahgan *et al*., 2007). In particular, the tribe Coprini was found to be paraphyletic and monophyletic in the nucleotide and protein trees, respectively (Figure 2, Figure S1). Contradictory results are frequently reported for this tribe, which is monophyletic in analyses using COI and COII (Villalba et al., 2002), but commonly paraphyletic in studies using sufficient taxon sampling, for example, in a phylogeny of nuclear (18S rDNA, 28SrDNA, CAD, and topoisomerase I) and mitochondrial genes (16S and COI) (Tarasov and Dimitrov, 2016).

In the present study, the phylogenetic relationships between Scarabaeinae tribes obtained with the two approaches (amino acid and nucleotide sequences) were: (Onthophagini + Oniticellini) Onitini) Eurysternini) (Coprini+ Phanaeini) (Figure 2). This result is similar to the phylogeny obtained based on one nuclear gene (28S) and two mitochondrial genes (COI and rrnL) (Monaghan et al., 2007). The presence of one monophyletic clade composed of Onthophagini and Oniticellini has also been reported in morphological and molecular studies (COI and 28S genes) (Philips, 2016; Mlambo et al., 2015). On the other hand, the sister group of this clade differs between morphological and molecular studies, including Sisyphini + *Epirinus* and Onitini (as observed in the present study), respectively (Monaghan *et al*., 2007; Tarasov and Genier, 2015). This incongruity may be related to the lack of morphological synapomorphies characterizing these tribes, as suggested by Tarasov and Dimitrov (2016).

*Coprophanaeus ensifer* was clustered with another representative of this genus (*Coprophanaeus* sp.), which belongs to the tribe Phanaeini, and with species of the tribe Coprini (*Canthidium* sp. and *Dichotomius schiffleri*) (Figure 2). The phylogenetic proximity between these tribes has also been reported in other studies using morphological and molecular markers (Philips et al., 2004; Tarasov and Dimitrov, 2016). However, this result diverges from the hypothesis of Zunino (1983) based on genitalia traits that suggests Phanaeini to be a sister group of Onitini. Morphological analysis also revealed a closer proximity of Phanaeini to Eucraniini (Philips and Scholtz, 2004). Such positioning could not be verified in our study since there is no Eucraniinimi mitogenome sequenced so far.

In the present study, *C. ensifer* is part of the most basal clade of Scarabaeinae, which is formed by species of Ateuchini, Coprini and Phanaeini tribes (Figure 2). These species show a neotropical distribution, especially in South America (Edmonds and Zídek, 2010; Valois et al., 2017; Cupello, 2018), except for *Sarophorus* sp. (Ateuchini) which is most basal and is found mainly in South Africa (Frolov and Scholtz, 2003).

The geographic distribution of these species that form the most ancestral clade suggests with Scarabaeinae originated in Africa, as suggested by Sole and Sholtz (2010). After its origin, colonization in South America must have occurred by the common ancestor of *Sarophorus* sp. and species of Ateuchini, Coprini and Phanaeini tribes. Furthermore, these results do not suggest a fauna exchange between South America and Africa during the early evolution of Scarabaeinae, as suggested by Gunter et al. (2016). However, this may be related to a limited number of mitogenomes characterized in Scarabaeinae.

The characterization and analysis of the *C.ensifer* mitogenome showed a genomic organization and the number of genes similar to those from the majority of Coleoptera species analyzed. Spacers and gene overlaps were also observed in this mitogenome. Regarding phylogenetic uncertainties in Scarabaeinae, the phylogenetic results suggest proximity between the tribes Phanaeini and Coprini, and place the tribe Oniticellini proximity to Onthophagini and Onitini. Despite this, inconsistencies were observed between the protein and nucleotide phylogenetic trees. ​Therefore, additional data from nuclear markers is necessary to elucidate the phylogeny of the Scarabaeidae family. The analysis suggests the origin of the Scarabaeinae subfamily in Africa. The results of this study provide a basis for future phylogenetic analyses of *Coprophanaeus* in order to gain a better understanding of its evolutionary history.

## Supplementary material

The following online material is available for this article:

Table S1 - List of species used in the analyses and their respective NCBI accession number, tribe and subfamily.

Figure S1 - Phylogenetic reconstructions of the family Scarabaeidae based on nucleotide sequences of the mitochondrial genome.

Figure S2 - Comparison between phylogenies generated based on nucleotide sequences and concatenated protein sequences of mitochondrial genes

File S1 - GenBank file of *Coprophanaeus (M.) ensifer* mitogenome, accession number MW122514.
