# Supplementary material for: Mitogenome of Coprophanaeus ensifer and phylogenetic analysis of the Scarabaeidae family (Coleoptera)
Source: Genet Mol Biol. 2021 Aug 9;44(3):e20200417. doi: 10.1590/1678-4685-GMB-2020-0417 (PMC8361247; doi:10.1590/1678-4685-GMB-2020-0417)
Supplement: Table S1 - [file 1415-4757-GMB-44-3-e20200417-s1.pdf]

Genetics and Molecular Biology
https://doi.org/10.1590/1678-4685-GMB-2020-0417

Supplementary Material to "Mitogenome of *Coprophanaeus ensifer* and phylogenetic analysis of the Scarabaeidae family (Coleoptera)"

Table S1 - List of species used in the analyses and their respective NCBI accession number, tribe and subfamily.

Subfamily	Tribe	Species	NCBI access number
Aphodiinae	Aphodiini	*Aphodius foetens*	KX087240.1
Aphodiinae	Aphodiini	*Aphodius* sp.	JX412755
Aphodiinae	Aphodiini	*Oxyomus sylvestris*	KX087329.1
Cetoniinae	Cetoniini	*Leucocelis* sp.	JX412740
Cetoniinae	Cetoniini	*Protaetia brevitarsis*	KC775706
Cetoniinae	Trichiini	*Osmoderma opicum*	NC 030778.1
Dynastinae	Oryctini	*Cyphonistes vallatus*	JX412731
Lucaninae	Aegini	*Odontolabis cuvera fallaciosa*	MF908524.1
Lucaninae	Dorcini	*Dynodorcus curvidens hopei*	MF612067.1
Lucaninae	Dorcini	*Prosopocoilus gracilis*	NC 027580.1
Melolonthinae	Euchirini	*Cheirotonus jansoni*	KC428100
Melolonthinae	Leucopholini	*Asthenopholis* sp.	JX412755
Melolonthinae	Melolonthini	*Melolontha hippocastani*	KX087316.1
Melolonthinae	Melolonthini	*Polyphylla laticollis*	KF544959
Melolonthinae	Melolonthini	*Rhopaea magnicornis*	NC013252
Melolonthinae	Melolonthini	*Schizonycha* sp.	JX412739
Rutelinae	Adoretini	*Adoretus* sp.	JX412788
Rutelinae	Anomalini	*Phyllopertha horticola*	KX087335.1
Rutelinae	Rutelini	*Popillia japonica*	NC 038115.1
Scarabainae	Ateuchini	*Sarophorus* sp.	JX412735
Scarabainae	Coprini	*Canthidium* sp.	MG253260.1
Scarabainae	Coprini	*Dichotomius schiffleri*	NC_039689.1
Scarabainae	Coprini	*Xinidium* sp.	JX412829.1
Scarabainae	Eurysternini	*Eurysternus caribaeus*	KU739494.1
Scarabainae	Eurysternini	*Eurysternus foedus*	KU739455.1
Scarabainae	Eurysternini	*Eurysternus hamaticollis*	KU739493.1
Scarabainae	Eurysternini	*Eurysternus inflexus*	KU739492.1
Scarabainae	Oniticellini	*Drepanocerus kirbyi*	KU739491.1
Scarabainae	Oniticellini	*Euoniticellus fulvus*	KU739453.1
Scarabainae	Oniticellini	*Euoniticellus intermedius*	KU739490.1
Scarabainae	Oniticellini	*Helictopleurus quadripunctatus*	KU739489.1
Scarabainae	Oniticellini	*Liatongus militaris*	KU739488.1
Scarabainae	Oniticellini	*Oniticellus egregious*	KU739487.1
Scarabainae	Oniticellini	*Scaptodera rhadamistus*	KU739460.1
Scarabainae	Oniticellini	*Tiniocellus sarawacus*	KU739486.1
Scarabainae	Oniticellini	*Tiniocellus spinipes*	KU739485.1
Scarabainae	Oniticellini	*Tragiscus dimidiatus*	KU739454.1
Scarabainae	Onitini	*Bubas bison*	KU739470.1
Scarabainae	Onitini	*Bubas bubalus*	KU739469.1

Scarabainae	Onitini	*Cheironitis hoplosternus*	KU739450.1
Scarabainae	Onitini	*Heteronitis castelnaui*	KU739468.1
Scarabainae	Onitini	*Onitis alexis*	KU739467.1
Scarabainae	Onitini	*Onitis falcatus*	KU739466.1
Scarabainae	Onitini	*Onitis fulgidus*	KU739431.1
Scarabainae	Onthophagini	*Caccobius nigritulus*	KU739484.1
Scarabainae	Onthophagini	*Cleptocaccobius convexifrons*	KU739436.1
Scarabainae	Onthophagini	*Digitonthophagus gazelle*	KU739497.1
Scarabainae	Onthophagini	*Milichus apicali*	KU739481.1
Scarabainae	Onthophagini	*Phalops ardea*	KU739473.1
Scarabainae	Onthophagini	*Phalops barbicornis*	KU739457.1
Scarabainae	Onthophagini	*Phalops smaragdinus*	KU739495.1
Scarabainae	**Phanaeini**	***Coprophanaeus ensifer***	**MW122514**
Scarabainae	Phanaeini	*Coprophanaeus* sp.	KU739465.1
