# Supplementary material for: Mitogenome of Coprophanaeus ensifer and phylogenetic analysis of the Scarabaeidae family (Coleoptera)
Source: Genet Mol Biol. 2021 Aug 9;44(3):e20200417. doi: 10.1590/1678-4685-GMB-2020-0417 (PMC8361247; doi:10.1590/1678-4685-GMB-2020-0417)
Supplement: Figure S1 - [file 1415-4757-GMB-44-3-e20200417-s2.pdf]

Genetics and Molecular Biology
https://doi.org/10.1590/1678-4685-GMB-2020-0417

Supplementary Material to "Mitogenome of *Coprophanaeus ensifer* and phylogenetic analysis of the Scarabaeidae family (Coleoptera)"

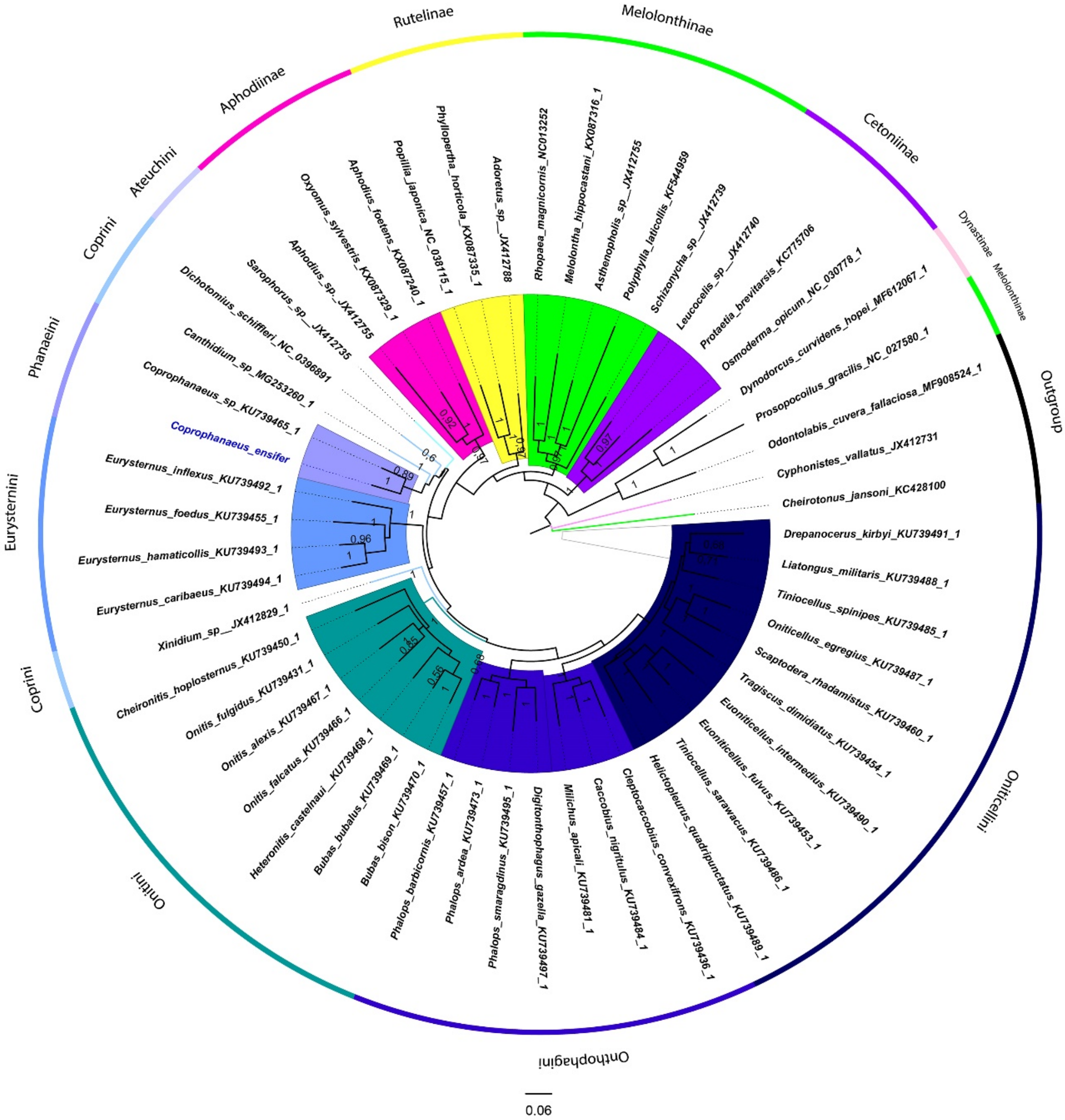

Figure S1 – Phylogenetic reconstructions of the family Scarabaeidae based on nucleotide sequences of the mitochondrial genome.
