# Supplementary material for: Mitogenome of Coprophanaeus ensifer and phylogenetic analysis of the Scarabaeidae family (Coleoptera)
Source: Genet Mol Biol. 2021 Aug 9;44(3):e20200417. doi: 10.1590/1678-4685-GMB-2020-0417 (PMC8361247; doi:10.1590/1678-4685-GMB-2020-0417)
Supplement: File S1 - [file 1415-4757-GMB-44-3-e20200417-s4.pdf]

Genetics and Molecular Biology
https://doi.org/10.1590/1678-4685-GMB-2020-0417

Supplementary Material to "Mitogenome of *Coprophanaeus ensifer* and phylogenetic analysis of the Scarabaeidae family (Coleoptera)"

LOCUS Coprophanaeus 14964 bp DNA circular INV 12-OCT-2020
DEFINITION ensifer Mitogenome.
ACCESSION Coprophanaeus
VERSION
KEYWORDS .
SOURCE mitochondrion Coprophanaeus ensifer
ORGANISM Coprophanaeus ensifer
Eukaryota; Metazoa; Ecdysozoa; Arthropoda; Hexapoda; Insecta;
Pterygota; Neoptera; Holometabola; Coleoptera; Polyphaga;
Scarabaeiformia; Scarabaeidae; Scarabaeinae; Scarabaeinae incertae
sedis; Coprophanaeus.
REFERENCE 1 (bases 1 to 14964)
AUTHORS Mello,C.A.A., Amorim,I.C., Silva,A.F., Medeiros,G.R., Wallau,G.L.
and Moura,R.C.
TITLE Direct Submission
JOURNAL Submitted (12-OCT-2020) Centro de Pesquisa do ICB, Instituto de
Ciencias Biologicas, Universidade de Pernambuco, Rua Arnobio
Marques, 310- Santo Amaro, Recife, Pernambuco 50.100-130, Brasil
COMMENT Bankit Comment: TOTAL # OF SEQS:1

##Assembly-Data-START##
Assembly Method :: Mitobim v. 1.9.1
Sequencing Technology :: Illumina
##Assembly-Data-END##
FEATURES Location/Qualifiers
source 1..14964
/organism="Coprophanaeus ensifer"

/organelle="mitochondrion"

/mol_type="other DNA"

/specimen_voucher="NM6645"

/db_xref="taxon:1269356"

/country="Brazil"

/lat_lon="-7.923283, -35.033329"

/collected_by="Amorim IC"

/identified_by="Amorim IC"

tRNA 1..66

/product="tRNA-Ile"

tRNA complement(64..132)

/product="tRNA-Gln"

tRNA 151..219

/product="tRNA-Met"

CDS 241..>1149

/codon_start=1

/transl_table=5

/product="NAD2 - NADH dehydrogenase subunit 2"

/translation="MFLFSLMIGTFISISSYSWLGMWLGLEINLLSIIPLLSKTNNSM
ASEAALKYFISQALASTLLLFSIIMMSLNLMYNINMNFYMLLIINTALLTKMGAAPFH
FWFPEVIEGLNWNNAFIMLTWQKIAPMILIMYTNMTMFYISIIIIISSTIGGFMGINQ
ISLRKILAYSSINHIAWMLAAMMFKETIWFYYFIIYSIISLNIIIIFKKLNIFYIKQL
FISMNNQPMMKFFFILNFMSLGGLPPFLGFMPKWLTIQTLIENNYFFITFMIIITTLI
TMYFYMRITLSTMVISMNELFYYKMPM"

tRNA 1232..1299

/product="tRNA-Trp"

tRNA complement(1292..1359)

/product="tRNA-Cys"

tRNA complement(1361..1426)

/product="tRNA-Tyr"

CDS 1447..>2961

/codon_start=1

/transl_table=5

/product="COX1 - Cytochrome c oxidase subunit 1"

/translation="MNKWLFSTNHKDIGTLYFMFGSWAGMVGTSLSLLIRAELGNPGT
LIGDDQIYNVIVTAHAFIMIFFMVMPILIGGFGNWLVPLMLGAPDMAFPRMNNMSFWL
LPPSLTLLLMSSMVENGAGTGWTVYPPLSANIAHSGASVDLAIFSLHLAGISSILGAI
NFITTVINMRSTGMTFDRMPLFAWAVVLTALLLLLSLPVLAGAITMLLTDRNLNTSFF
DPVGGGDPILYQHLFWFFGHPEVYILILPGFGMISHIISQESSKKETFGTLGMIYAMM
AIGLLGFIVWAHHMFTVGMDVDTRAYFTSATMIIAVPTGIKIFSWLATLHGTQLNYSP
SLIWALGFVFLFTVGGLTGVVLANSSIDIVLHDTYYVVAHFHYVLSMGAVFAIMAGFV
HWFPLFTGLTMNNKFLKIQFLIMFIGVNMTFFPQHFLGLSSMPRRYSDYPDAYMTWNV
ISSIGSMISLVSIFLFLFIIWDSFTSMRKSIGTLSMNTSIEWMQLMPPAEHSYNE"

tRNA 2986..3050

/product="tRNA-Leu"

CDS 3078..>3716

/codon_start=1

/transl_table=5

/product="COX2 - Cytochrome c oxidase subunit 2"

/translation="QDAASPLMEQLIFFHNHALLILLMITVTVTYLMSTLFFNKYNYR
FLLEGQTIEIIWTIIPAVTLIFIALPSLQLLYLLDEINNPLVSVKTIGHQWYWSYEYS
DIKKIEFDSYMIPTNDLPLNGFRLLDVDNRTILPYNTQIRMLVTAADVIHSWTIPSLS
VKIDATPGRLNQISFLMNRAGLFFGQCSEICGANHSFMPIVVESISINYFIKW"

tRNA 3730..3801

/product="tRNA-Lys"

tRNA 3808..3874

/product="tRNA-Asp"

CDS 3875..>4027

/codon_start=1

/transl_table=5

/product="ATP8 - ATP synthase subunit 8"

/translation="MPQMAPLNWLMLFIYFCFIFLLFNTMNYYLFNYKIKTYTFKKIK
TLINWKW"

CDS 4024..>4689

/codon_start=1

/transl_table=5

/product="ATP6 - ATP synthase subunit 6"

/translation="MMTNLFSSFDPGTYLNTSLNWLSTLLGLLFLPTMYWLIPSRYNF

LWNKIILTLHNEFKILLGNNKIKGSTLIFISLFSMIMFNNFLGLFPYIFTSTSHLILT

LTLALPLWLSFMIYGWFNHTMHMFTHLVPQGTPAVLMPFMVCIETISNVIRPGTLAVR

LAANMIAGHLLLTLLGNTGSMLNMIMINFLIMTQLLLLMLESAVAIIQSYVFAVLSTL

YSSE"

CDS 4698..>5480

/codon_start=1

/transl_table=5

/product="COX3 - Cytochrome c oxidase subunit 3"

/translation="MSKKNHPYHLVDASPWPLLGALSAMVTMIGIIKWFHFYNYNLFM

LGMLSMFMIMYQWWRDVVREGTFQGLHTYPVTMGLRWGMILFITSEVFFFISFFWGFF

HSSLSPSIELGMMWPPKGITPFNPLQIPLLNTLILLTSGLTVTWAHHSLIENDFNQTT

QGLTLTVLLGIYFTMLQAYEYIEAPFTIADSVYGSSFFMATGFHGLHVIIGTTFLAVC

LFRHLNNHFSCIHHFGFEAAAWYWHFVDVVWLFLYISIYWWGS"

tRNA 5491..5555

/product="tRNA-Gly"

CDS 5574..>5903

/codon_start=1

/transl_table=5

/product="NAD3 - NADH dehydrogenase subunit 3"

/translation="MSMIIYIICMIMMLLSTILSKKSFMDREKSSPFECGFDPKSSAR

MPFSLQFFLIAVIFLIFDIEVALLIPMILTLNISNIMNYVMIIFFFLFILLLGLYHEW

NQGALNWA"

tRNA 6038..6105

/product="tRNA-Ala"

tRNA 6108..6174

/product="tRNA-Arg"

tRNA 6180..6247

/product="tRNA-Asn"

tRNA 6248..6316

/product="tRNA-Ser"

tRNA 6317..6383

/product="tRNA-Glu"

tRNA complement(6382..6448)

/product="tRNA-Phe"

CDS complement(<6456..8111)

/codon_start=1

/transl_table=5

/product="NAD5 - NADH dehydrogenase subunit 5"

/translation="MSFMLSLNFMILDYSLFLEFEVLSINSCSILMSILLDWMSLLFM
SFVLFISSMVIFYSEEYMEGDLCINRFIMLVSMFVLSMMLLIISPNLISILLGWDGLG
LVSYCLVIYYQNIKSYNAGMITALSNRIGDVALLLAIAWMFNYGGFNYYFYVNYIHND
NLNLISMLIVLAAMTKSAQIPFSSWLPAAMAAPTPVSSLVHSSTLVTAGVYLLIRFNF
ALDNKIMMFLLFIGSMTMFMAGLGASFEFDLKKIIALSTLSQLGLMMSILALGEYNLA
FFHLLTHALFKALLFMCAGCMIHNLGNCQDIRFMGGLINLMPLTCSFFIISNMSLCGL
PFLAGFYSKDLILEVLSMNYLNIFIYIIFFISTGLTVAYTFRLIYYVIVGDFNYFSLN
MISDNGFIMLKGMSGLILLVIFGGSMLSWLMFPIPYFICLPLFMKMLALMVTILGGLL
GYEISKFSLCYTLKSLNNLKLSLFFSSMWNMPFISTFGVNYYPLIMGNMIYKNIDQGW
SEYLGAQNIYYNIKNSSLFLQILYNNNLKIFLLLLVLWVIFMMI"

tRNA complement(8160..8226)

/product="tRNA-His"

CDS complement(<8257..9534)

/codon_start=1

/transl_table=5

/product="NAD4 - NADH dehydrogenase subunit 4"

/translation="MMKFCLMMLFMIPLSFKSKSFWLNQYLYFVMFFLFLLSFSYNYM
FKNISYFLGCDLLSFVMMLLSYWICALMLMASESVYYKNYHYNLFMLVILLLLISLFF
TFISMNLFVFYLFFEMSLIPTLILIIGWGYQPERLQAGIYLLFYTLLASLPMMISIFY
YYSEFNSLNFFFLYNMVNNIYMYLCMNVVFLVKMPMYLLHLWLPKAHVEAPVSGSMIL
AGVMLKLGGYGLMRLMMIFMKLGIKINFIFIVISMFGGVLVSLICLRQMDIKSLIAYS
SVAHMGLVLGGIMTMSYWGMCGALVMMLAHGLCSSGLFCLANINYERLLTRSLYLNKG
LINLMPSMSLWWFLLSSSNMAAPPSLNLLGEIMLINSLVSWNYLCMLMLMFLSFFSAA
YSLYLYAYTQHGKLFSGLYSFSSGLIREYLLLFL"

CDS complement(<9534..9788)

/codon_start=1

/transl_table=5

/product="NAD4L - NADH dehydrogenase subunit 4L"

/translation="MMYFSGLISFTMKRKHLLLMLLSLEFIILSLYFNMFLYLSYFNF
EFFFGMVFLTMSVCEGALGLSILVSLIRTHGNDYFQTFSVL"

tRNA 9827..9892

/product="tRNA-Thr"

tRNA complement(9893..9959)

/product="tRNA-Pro"

CDS 9971..>10453

/codon_start=1

/transl_table=5

/product="NAD6 - NADH dehydrogenase subunit 6"

/translation="MLMSICLLMSLYMIFLKHPLSMGITLLVQTIMISLTMGFFNLNY

WYSYILFLVMIGGMLVLFIYMTSIASNEMFYPSIKLFIISILIMNLMLFIFMNLDTYY

FMMNNFYENNMYQNNFNLSLNKYLNSPNNSIMCMLIIYLLITLVAIVKITNFKMGALR

Q"

CDS 10478..>11581

/codon_start=1

/transl_table=5

/product="CytB - Cytochrome b"

/translation="MRKMSPLLKLINNSLIDLPTPSNISTWWNFGSLLGLCLMIQIVT

GIFLAMHYTPDIDMAFNSVIHICRDVNYGWLIRTLHANGASFFFICLYAHVGRGLYYS

SYNLHMTWTMGVLILFAVMATAFLGYVLPWGQMSFWGATVITNLLSAIPYVGNMIVQW

LWGGFAVSNATLTRFFALHFLLPFIVAAMVMIHLMFLHQTGSNNPLGTNSNIDKSPFH

PYFSLKDSVGFIILLMSLMILVLTNPYMLSDPDNFMPANPLVTPIHIQPEWYFLFAYA

ILRSIPNKLGGVIALVLSIAILLIMPFTNKKMMQSTQFYPINKIMFWTFFNIVILLTW

IGARPVEDPYIMIGQILTIAYFLYYMLNPLLSML"

tRNA 11610..11677

/product="tRNA-Ser"

CDS complement(<11739..12632)

/codon_start=1

/transl_table=5

/product="NAD1 - NADH dehydrogenase subunit 1"

/translation="MLSLISSLILIICVLVGVAFLTLLERKVLGYIQIRKGPNKVGYM

GIPQPFSDAIKLFTKEQTFPYMSNFIIYYMSPVFSLFLSLLLWLCMPFLTVLFNFNLG

ILFFLCCSSLGVYTVMIAGWSSNSNYSMLGALRAVAQTISYEVSLFLILLSFLYLISS

LNMLNLMKYQEYMWFMFLCLPLCMMWFVSSLAETNRTPFDFAEGESELVSGFNVEYSS

GGFALLFLAEYSSILFMSMLCCLLFLGGNIMSVFFYMKLVFMSFFWIWVRGTLPRYRY

DKLMYMCWKSFLPVSLNYLFFF"

tRNA complement(12651..12719)

/product="tRNA-Leu"

rRNA complement(12683..14053)

/product="23S ribosomal RNA"

tRNA complement(14051..14121)

/product="tRNA-Val"

rRNA complement(14122..14964)

/product="16S ribosomal RNA"

BASE COUNT 5939 a 1987 c 1420 g 5618 t

ORIGIN

1 aatgaagtgt ctgaataaag aattattttg atagaataaa acatgtgact attttcacct

61 tcattataat taatagaatt aaactatttc tttaaatatc aaaaattcat gtacatctta

121 tactaaatta tatttattaa tttttttaat agaaagataa gctaattaag ctattgggtt

181 catacccccag ctataaaggt taaaatcctt ttctttttga tttttatctt ctataaatta

241 atatttttat tttctctaat aattggtact tttatttcaa tttcatctta cagatgatta

301 ggaatatgat taggattaga gattaattta ttatcaatta ttcctttact tagaaaaaca

361 aataattcta tagcttcaga agctgcctta aaatatttta ttagacaagc tttagcttca

421 acattattat tatttagaat tattataata tcattaaatt taatatataa tattaatata

481 aatttttata tactattaat tattaatact gctctattaa ctaaaatagg tgcagcacca

541 tttcatttct gattccccga agtaattgaa ggtcttaatt gaaataatgc atttattata

601 ttaacttgac aaaaaattgc accaataatt ctaattatat atactaatat aactatattt

661 tatatttcaa ttattattat tattagctct actattgggg gatttatagg aattaatcaa

721 attagattac gaaaaatttt agcttattcc tcaattaacc atattgcttg aatattagct

781 gcaataatat ttaaagaaac aatttgattt tattatttta ttatttactc tatcatttct

841 ttaaatatca ttattatttt taaaaaacta aatatctttt atattaaaca attatttatt

901 tcaataaata accaacctat aataaaattt ttttttatct taaatttcat atctttaggg

961 ggattacccc cttttttagg ttttatacca aaatgattaa caattcaaac tttaattgaa

1021 aataattatt tctttattac atttataatt atcattacaa cattaattac aatatatttt

1081 tatatacgaa ttacattaag tacaatagtt atttccataa atgaattatt ttactacaaa

1141 atgcctataa taaatataaa ttgaatttat agaattaatt ttattactat tataagatta

1201 atttttatta caattatatt taattttgat taaggattta agttaaatta aactagaaac

1261 cttcaaagtt tcaaatagag acagtatctt taagccttag aaattaaatt tctactttaa

1321 atttgcaatt taacatcatt ttttattgac tataagactt taataaagga atattattat

1381 tcgttaataa atttacaatt tatcgcttaa actcagccac tttactactt tactactttt

1441 ttaaaaatga ataaatgatt attttcaaca aatcataaag atattggaac tttatacttc

1501 atattcggaa gatgagcagg aatagtaggc acatcactta gtttattaat tcgagctgaa

1561 ttaggaaacc ctggtacact aattggtgat gatcaaattt ataatgttat tgtaacagct

1621 catgctttta ttataatttt tttcatagta atacctattt taattggtgg atttggtaac

1681 tgacttgtac ctttaatatt aggcgctcct gatatagcat tccctcgtat aaataatata

1741 agattttgac tactaccccc ttcattaaca ttactattaa taagaagaat agttgaaaat

1801 ggggctggaa caggatgaac agtttaccct cctctttcag ctaatattgc ccatagtgga

1861 gcttcagtag atttagctat ttttagatta catttagcag gtatttcttc aattcttggt

1921 gctatcaatt ttattacaac agtaattaat atacgatcaa caggaataac ttttgatcga

1981 atacctttat ttgcttgagc agtagtatta actgctttat tattactttt atctttacca

2041 gttttagctg gagcaattac tatgctttta actgatcgaa atttaaatac atcattcttc

2101 gatcctgttg gaggcggaga cccaattcta taccaacatt tattttgatt ttttggacat

2161 ccagaagttt atattttaat tttacctgga tttggaataa tttctcatat tattagtcaa

2221 gaaagaagaa aaaaagaaac atttggaact ttaggtataa tttatgcaat aatagcaatt

2281 ggattactag gttttattgt atgagcacac catatattta cagtaggtat agatgttgac

2341 actcgagctt attttacttc tgctactata attattgcag ttcccactgg aattaaaatt

2401 tttagatgat tagctacatt acatggtact caattaaatt attctccctc tttaatttga

2461 gctttaggat ttgtattttt atttactgtc ggtggattaa ctggtgttgt attagcaaat

2521 tcttctattg atattgtttt acatgatact tattatgttg ttgctcattt tcactatgtt

2581 ttatcaatag gagcagtatt tgcaattata gcaggatttg tccattgatt tcctttattt

2641 acaggtctta ctataaataa taaattttta aaaattcaat ttttaattat atttattgga

2701 gttaatataa catttttccc acaacatttt ctaggattaa gaagaatacc tcgccgatat

2761 tcagactacc ctgatgctta tataacatga aatgtaattt catctattgg gtctataatt

2821 tctttagtaa gaattttcct atttttattt attatttgag atagatttac ttcaatacga

2881 aaatcaattg ggactctaag aataaataca tctattgaat gaatacagct tataccccca

2941 gcagaacata gctataatga attacctatt ttaactaatt aattttctaa tatggcagat

3001 tagtgcggtg aatttaagct tcacatataa agtttacgct ttttttagaa attgctactt

3061 gaaaaagaac tttcctccaa gatgccgctt ctccacttat agaacaatta attttctttc

3121 ataatcatgc tttattaatt ttattaataa ttactgtcac agtaacttat ttaataagaa

3181 ctttattttt taataaatat aattatcgat ttcttttaga aggacaaact attgaaatta

3241 tttgaacaat tattccagca gtaacattaa tttttattgc attaccttca ttacaattac

3301 tttatttact tgatgaaatt aataaccctt tagtatcagt aaaaactatt ggacatcaat

3361 gatattgatc ttatgaatat tctgatatca aaaaaattga atttgattct tatataattc

3421 ctacaaatga tctaccttta aatggatttc gccttttgga tgttgataac cgaaccattc

3481 ttccttataa tacacagatt cgaatattag taacagcagc tgatgttatt cattcatgaa

3541 ctatcccctc actaagagta aaaattgatg caacacctgg acgacttaat caaattagat

3601 tcttaataaa tcgagcagga ttattttttg gtcaatgttc tgaaatttgc ggggcaaatc

3661 atagttttat acctattgta gtagaaagaa tttcaattaa ttattttatt aaatgaatta

3721 ataaaacatc attagatgac tgaagtgcaa gtattggtct cttaaaccac cgtatagtaa

3781 tctagcaatt acttctaatg aaataaaaaa aatttagtta aacacaataa cattagcttg

3841 tcaagctaaa attattcatg aagaataatt tttaatccct caaatagcac cattaaattg

3901 attaatatta ttcatttatt tttgtttcat tttcttactt tttaatacaa taaattatta

3961 tttatttaat tataaaatta aaacttatac ttttaaaaaa attaaaactt taattaattg

4021 aaaatgataa caaatttatt ttcttcattt gacccaggca catatttaaa tacatcctta

4081 aattgattaa gaacattatt agggctttta tttttaccta caatatactg attaattcct

4141 tctcgttata attttctgtg aaataaaatc attttaactt tacataatga atttaaaatt

4201 ttacttggta ataataaaat taagggaaga acattaattt ttatttcatt attttcaatg

4261 attatattta ataatttttt agggttgttt ccttatattt ttactagaac aagacattta

4321 attttaactt taaccttagc attacctctt tgattaagat ttataatcta tgggtgattc

4381 aatcatacaa tgcatatatt tacacattta gtgcctcaag gcacaccagc agtgcttatg

4441 ccatttatag tatgtattga aactattagt aatgtaattc gacctggaac attagcagta

4501 cggttagcag cgaatataat tgcaggccat ttattactta ctttattggg aaatacaggg

4561 tcaatactaa atataattat aattaatttt ttaattataa ctcaactatt attattaata

4621 ttagaatcag cagttgctat tattcaatct tatgtgtttg ctgtattaag aacattatat

4681 tctagtgaag taaactaatg tcaaaaaaaa atcaccctta ccatttagtt gatgctagac

4741 cttgaccctt attaggcgca ttaagagcta tagtaacaat aattggaatt attaaatgat

4801 ttcattttta taattacaat ttatttatat taggtatatt atctatattt ataattatat

4861 accaatgatg acgagatgtt gtccgagaag gaacattcca aggattacat acttaccctg

4921 ttactatagg tttacgatgg ggtataattt tatttattac atcagaagta tttttttca

4981 tttctttttt ttgaggattt tttcatagaa gactatctcc atctattgaa ttaggtataa

5041 tatgaccccc caaaggaatc acacctttca atccattaca aattccttta ttaaatacct

5101 taattttatt aacatcagga ttaacagtta catgagctca tcatagttta attgaaaatg

5161 attttaacca aactactcaa ggattaactt taactgtatt attaggtatt tattttacaa

5221 tattacaagc ttatgaatat attgaagctc cttttacaat tgcagataga gtatacggtt

5281 catctttctt tatagcaact ggatttcatg gtcttcacgt tattattgga actacatttt

5341 tagctgtttg tttatttcgt catttaaata accatttttc atgtattcat cattttgggt

5401 ttgaagctgc agcatgatat tgacattttg ttgatgttgt atgattattc ctttatattt

5461 ctatctattg atgaggtaga taattaatct atttgtatag tataaaaatt ataattgatt

5521 tccaatcaaa agatctaaac attttagtat aaataattac aataattttt tatatatcaa

5581 taattattta tattatttgt ataattataa tattattatc aactatccta tcaaaaaaaa

5641 gatttataga tcgagaaaaa agatcaccat ttgaatgtgg atttgatcct aaaagatcag

5701 ctcgaatacc tttttcttta caattttttt taattgcagt aattttttta atttttgata

5761 ttgaagttgc tttattaatt cccataattt taacattaaa tatttcaaat attataaatt

5821 atgttataat tatttttttt ttttattta ttttactatt agggttatat cacgaatgaa

5881 atcaaggggc tttaaactga gctaattaat tagggtaata gttaaatatt tcaaatatta

5941 taaattatgt tataattatt ttttttttt tatttatttt actattaggg ttatatcacg

6001 aatgaaatca aggggcttta aactgagcta attaattagg gtaatagtta aaaataacat

6061 ttaagttgca tttaaaaagt attgattaat aatcaattta ccttactaaa taagaaacaa

6121 attattgtat ttagtttcga cctaaaattt tggtgataat tacaccctta tttacatatt

6181 taattgaagc caaaatagcg gcatatcact gttaatgata atattggatt tttacaaccc

6241 aattaaaaag ataagattaa atcagaatta agcttctaac ttaaattctt agcagtgaaa

6301 ctctgttaat atctttattt atatagttta aataaaacat tatattttca ttataaaaac

6361 agaattaatt tttcttataa atacctaaaa atatgactat taccctaata tcttcaatat

6421 tatgctctaa actttaagct atttaagtaa aatataatta ttataaaaat aactcataaa

6481 actaataata ataaaaaaat ctttaaatta ttattatata aaatttgtaa aaataaagaa

6541 gaattcttaa tattatagta aatattttga gcgcctaaat attctgatca accttggtca

6601 atatttttat aaattatatt tcctataatt agaggataat aattaacgcc aaaagtagaa

6661 ataaagggca tatttcatat tgaagaaaaa aataagctta actttaaatt atttaaagac

6721 tttaaagtat aacataaaga aaattttgaa atttcatacc ctaataaacc acctaaaatt

6781 gtcactatta atgctaatat ttttataaat aaaggtaaac aaataaaata aggaataggg

6841 aatattaatc atctcaatat acttccacca aaaattacta ataaaattaa acctcttatc

6901 ccctttaata taataaatcc attatctgaa attatattta aagaaaaata attaaaatca

6961 ccaacaatta cataataaat taaccgaaaa gtatatgcta ctgttaaccc tgtggaaata

7021 aaaaaaataa tataaataaa aatatttaaa taatttattg ataaaacttc taaaattaaa

7081 tcctttgagt aaaaccctgc taaaaatggt aaaccacata atgatatatt agaaataata

7141 aaaaatgaac aagttaaagg tattaaatta attaaacccc ccataaatcg aatatcttga

7201 caattcccta aattatgaat tatacatcca gcacatataa ataataaagc tttaaataat

7261 gcatgagtta ataaatgaaa aaaagctaag ttatactcac ctaaagctaa aattcttatt

7321 attaaaccta attgacttaa agttgataaa gcaataattt tttttaaatc aaattcaaat

7381 cttgcccсta aaccagctat aaatatagtt attctaccaa taaataataa aaatattata

7441 atcttattat ctaaagcaaa attaaaacga attaataaat atactcccgc agtaactaaa

7501 gttgacgaat gtactaatga agatacagga gtaggtgctg ctatagctgc aggtaatcaa

7561 gaagaaaatg gaatttgagc agacttagtt attgcggcta atacaattaa tatactaatt

7621 aaatttaaat tatcattatg aatataatta acataaaaat aataattaaa tccaccataa

7681 ttaaatattc aagcaatagc taataataaa gctacatccc caattcgatt tcttaaagca

7741 gtaattatac ctgcattata agatttaata ttttgataat aaattactaa gcaataagaa

7801 actaatccta acccatctca tcctaataaa attctaatta aatttggcct aataattaat

7861 aatattattg atagcacaaa tattgatact aatataataa aacgattaat acacaaatca

7921 ccttctatat attcttcact ataaaaaatt actatagatg aaataaataa aacaaatctt

7981 ataaataata aagatattca atctaataaa attcttatta aaatactaca agaattaatt

8041 cttaaaactt caaattctaa gaataatcta taatctaaaa ttataaaatt taatcttaat

8101 ataaaactca taatactaaa aaataaaaag aaaccaaaat aaataataca aattgaaatt

8161 atctaaggta ttattttaat acatctatga ttccacaaat caatatttta tttaaactac

8221 ttaaattttt aaattcacaa tgtaaaatat tcactgtaaa aataataata aatattctcg

8281 aattaatcct ctagaaaaag aatataaccc agaaaataac ttaccatgtt gagtataagc

8341 atataaatat aatgaataag cagcactaaa aaaagataaa aatattaaca tcaatataca

8401 taaataattt caactaacta aactattaat taatataatt tcaccaagta aatttaaaga

8461 aggtggtgca gctatattag acctacttaa taaaaatcat cataaactca ttgatggtat

8521 taaattaatt aaacccttat ttaaatataa tctccgagtt aataatcgtt cataattaat

8581 atttgctaaa caaaataatc cagaagaaca taatccatgc gctaatatta ttactaatgc

8641 gccacatata cctcaataag atatagttat aatcccgcca agaacaagcc ctatatgagc

8701 aactgaagaa taagcaatta aagatttaat atctatttga cgtaaacaaa ttaatgaaac

8761 taaaacacct ccaaatattc taataacaat aaaaataaaa ttaattttaa ttcctaattt

8821 tataaaaatt attattaatc gcattaaacc atatccacct aattttaata ttactccagc

8881 taaaattatt gaaccagata caggtgcttc aacatgggcc ttaggtaatc ataaatgtaa

8941 taaatatatt ggtattttaa ctaaaaaaac aacatttata cataaatata tataaatatt

9001 attaactata ttatataaaa aaaaaaaatt taaactatta aattcactat aataataaaa

9061 aattgaaatt attataggta atgaagctaa taaagtataa aataataaat aaatccctgc

9121 ttgtaaacgt tctggctgat accctcaacc aataattaaa attaatgtgg gaattaaact

9181 tatttcaaaa aataaataaa aaacaaataa atttatagaa ataaaagtaa aaaataaaga

9241 aattaataat aataaaataa ccaatataaa taaattataa tgataatttt tataataaac

9301 tgattctctt gctattaata ttaatgcaca aattcaatat cttaataata ttataacaaa

9361 agaaagtaaa tcacatccta aaaaatatga aatattctta aatatataat tatacctaaa

9421 acttaaaaga aataaaaaaa atattacaaa atataaatat tgatttaatc aaaaactttt

9481 agacttaaaa cttaatggaa tcataaataa tattattaaa caaaatttta tcataaaact

9541 ctaaatgttt gaaaataatc attaccatga gttcgaatta aagaaactaa aattgataaa

9601 cctaatgctc cttcacaaac tcttatagtt aaaaatacta taccaaaaaa aaattcaaaa

9661 ttaaaatatc ttaaatataa aaatatatta aaatataaac ttaaaataat aaactctaat

9721 cttaataata ttaataataa atgtttacgt tttatagtaa aagaaattaa cccagaaaaa

9781 tatataatta cagaaaatac taaacaaaaa attaacatta attaaagttt taataattta

9841 tacataaaat atcggtcttg taaaccgaaa aaaagaaaaa tcttttaaaa cttcagagaa

9901 agaataatca ttccatcatt aatctccaaa attaatattt taacttaaac tattctctga

9961 tatcttattc atactaatat caatttgttt actaatatct ttatatataa tttttttaaa

10021 acatccttta tcaataggaa tcactttatt agtacaaaca attataattt cattaactat

10081 gggatttttt aatctaaatt actgatattc ttacatttta tttcttgtaa taattggtgg

10141 aatattagtt ttattcattt atataactag tattgcttca aatgaaatat tttatccttc

10201 aatcaaatta tttattattt ctattttaat tataaattta atattgttta ttttcataaa

10261 tttagatact tattatttta taataaataa tttttatgaa aataatatgt atcaaaataa

10321 ttttaactta tctttaaaca aatatttaaa ttcacctaat aattctatta tatgtatatt

10381 aattatttac ttattaatta ctttagtagc aatcgtaaaa attactaatt ttaaaatagg

10441 agctttacga caaataaatt aactaatgaa aacaccaata cgaaagatat cacccctact

10501 aaaattaatt aataattctt taattgatct accaacacct tctaatatta gaacatgatg

10561 aaattttggt tcattattag gtctatgttt aataattcaa attgtaacag gaattttttt

10621 agctatgcac tatacaccag atattgatat agcttttaat agagtaattc atatttgtcg

10681 tgatgtaaat tatgggtgat taattcgtac tttacatgct aatggggcaa gatttttctt

10741 tatttgttta tatgctcatg taggacgagg cttatattat tcatcttata acttacatat

10801 aacttgaact ataggggttc ttattttatt cgcagtaata gcaacagctt tccttggata

10861 tgttttacct tgaggtcaaa tatcattttg aggggcaaca gtaattacaa atttattatc

10921 cgcaatccct tacgtaggca atataattgt acaatgacta tgaggggggct ttgcagtaag

10981 aaatgcaact ttaactcgat tttttgcttt acattttctt ttaccattta ttgttgctgc

11041 aatagtaata attcatctta tatttttaca tcaaacaggt tcaaataatc ctctaggaac

11101 aaatagtaat attgataaat ctcccttcca tccttatttt tctttaaaag actcagtagg

11161 atttattatt ttattaatat cattaataat tttagtactc acaaatccgt atatattaag

11221 tgatcctgat aattttatac cagcaaatcc tttagtaact ccaattcata ttcaacctga

11281 atgatacttc ttatttgcat acgcaatctt acgatcaatt cctaataaat tagggggcgt

11341 aattgcatta gtattatcaa ttgctatttt attaattata ccatttacaa ataaaaaaat

11401 aatacaaagt actcaatttt acccaattaa taaaattata ttttgaactt tctttaatat

11461 tgttatttta ttaacttgaa ttggagcccg accagtagaa gatccttata ttataattgg

11521 acaaatttta acgattgcct atttttata ttacatatta aatcccttat tatcaatatt

11581 atgagattta atcattttca aaaacaatta attaatgaac ttgtaaaagt gtatactttg

11641 aaagtataaa aaagagattt aaattctcta ttaatttttt tggtactaaa ttttattaac

11701 tacattaaaa ttgcaaataa aaataatttt aatccccсаа aaaaaaataa ataatttaaa

11761 gaaacaggta aaaacctttt tcaacatata tatattaact tatcatatcg ataccgaggt

11821 aaagtccccc gcactcaaat ccaaaaaaaa gatataaata ctaactttat ataaaaaaat

11881 actcttataa tattcccacc taaaaataac aaacaacata atattcttat aaataaaatt

11941 cttgaatatt cagctaaaaa taataatgca aagccacctc ttctatattc tacattaaat

12001 cctgatacta attctgattc cccctctgca aaatcaaagg gagtacgatt agtttctgct

12061 aacctagaaa caaatcatat tatacataaa ggtaagcata aaaatataaa tcatatatat

12121 tcttgatatt ttattaaatt taatatattt aatctagaaa ttaaatataa aaaagataat

12181 aaaattaaaa ataatctaac ttcatacgaa attgtctgag caacagcccg caatgcccct

12241 aatattgaat aattagaatt agatgatcac cccgcaatca taacagtata aacccctaaa

12301 ctagaacaac ataaaaaaaa taaaataсct aaattaaaat taaataaaac tgtcaaaaat

12361 ggcatacata atcataataa taaagataaa aataaactaa aaacaggaga tatataataa

12421 ataataaaat ttgatatata aggaaaagtt tgttccttag taaataattt aatagcatca

12481 ctaaaaggct gtggaattcc tatataaccc actttattag gacccttacg aatctgaata

12541 taacctaata ccttacgctc taataaagtt aaaaaagcta cacctactaa aacacaaata

12601 attaaaatca acctagaaat taatcttaaa ataacatcct taattaaaat tattacttat

12661 attaatatat taatatatgt ttaaattcta agtttaaagc actagtctgc caaagtaata

12721 tttaaattat taatattctt actataataa ttattacgaa tatttggtcc tttcgtacaa

12781 aaatattcta atttttaaa gatagaaacc aacctggctc acgccggttt aaactcagat

12841 catgtaaaat tttaaaggtc gaacagacct aatcctttta gttgctgcgc caaaagtaaa

12901 ttttaatcca acatcgaggt cgcaaactct tttttcgata agaactctaa aaaaaaattac

12961 gctgttatcc ctaaggtaat ttaatctttt aatcattaac tatggatcac ttaatcatat

13021 atcaatggta taatttaaaa aaaattactt taatttttta attgccccaa ccaaatataa

13081 tttaatataa tcgaatctaa aattctttaa ctttcaaaaa tactaaatta tataaaactc

13141 tatagggtct tctcgtcttt taaaaatatt taagcttttt tacttaaaga taaaattcta

13201 atttaaatta aattgagaca gtctttttct cgttcgacct ttcataccag ttcccaatta

13261 aaaaactaat tattatgcta cctttgcacg gtcagagtac cgcagccatt taaaattaac

13321 ttattcatag ggcaggtcag actttatatt ataagcaaaa agacatgttt ttaataaaca

13381 ggcggaaaaa tgctttgccg agttccttaa tttaaccttg gtcaataaat taaaattaca

13441 tttaattata ctaattttat cattattaca tatattttta tattaaataa ataattttaa

13501 taaaaaactt aaaattctat ataaatctaa tatacataaa aatttgttat aacacaattt

13561 aagtaaaaaa aacttccaat cctattaatt tttatatatt taaattaatt tttaatatt

13621 tattttaaag cttatcccct aaaatattaa ttaattatat taatataaat aaagaattat

13681 tttaattata ataattaact aaattaaatt tatttcttaa aaaactagat aaatttaaaa

13741 acgaataacg tttcattact aaaaaattat tttaaatatt tatgctacaa taaaatttat

13801 aattttttag ctcttttaaa ttcgagaaaa ttaaatttca taattattat ttaataaacc

13861 ctgataccca aggtacaaaa aattaatttt tcttttaaaa atttcaattt tcataatatt

13921 taaatcattc aatcacttta ctgattaact ataattcata aaaattttat ctaaattaat

13981 aaataaatat aaaaatattt tttttaaata tttaaataaa ttaataatat ataaaataaa

14041 ctattagatt tcaaattaaa ttgatttaca caactaactc cttagtgtaa gtaagatact

14101 ttttaaacaa gctctaattt gtactaacca ggtacacctt ccggtacacc tactatgtta

14161 cgacttattc caccttaggg ttcggtggaa gcgacgggcg atatgtgcat attctagagc

14221 tatagtcata cgttttaagt taaacatatt acttccaaat ccattttatg caataatttt

14281 aattattaca accatataaa taaattcatt gtaacccatc tctcctcaat tataggctgt

14341 atcttgatct gatttaattc aaatttttga cagatgaaca ttccgcaaga ccttttaaac

14401 atattcgact acgacgatat acaaactagt aaattaagta tgattaatcg tggatcatcg

14461 attataggac aggttcctct gagaaggcta aaataccgcc aaatctttcg aatttcaagg

14521 gcataactac tactgctcag gcattttctt ttatattctt tataataggg tatctaatcc

14581 tagtctaata ttagaatctc acaactccag tttttaaaat taacttaact taatttaaat

14641 ttctaatttc actcattaaa tttaatttta aattaaactt taaattatac ctagtattaa

14701 ccgaataaat ttaacggcat catctgttta accacggctg ctggcacaaa tttagccaat

14761 actcttatat atttctccat caaaattccc tttattgtaa aagactcatt attgcatgat

14821 ttatctaccc ataagactgt cggctaaaaa attattattt aaataaaaat tctaattgcc

14881 atcaagattt acaattcgta caaaaaaatt cgcatataac tgtgaaaata tatattaaat

14941 ttataaacta aaataaaatt ttaa

//
